# Antileukemic Natural Product Induced Both Apoptotic and Pyroptotic Programmed Cell Death and Differentiation Effect

**DOI:** 10.3390/ijms222011239

**Published:** 2021-10-18

**Authors:** Wohn-Jenn Leu, Hsun-Shuo Chang, Ih-Sheng Chen, Jih-Hwa Guh, She-Hung Chan

**Affiliations:** 1 School of Pharmacy, National Taiwan University, No.33, Linsen S. Rd., Zhongzheng Dist, Taipei 100025, Taiwan; d02423201@ntu.edu.tw (W.-J.L.); jhguh@ntu.edu.tw (J.-H.G.); 2 School of Pharmacy, College of Pharmacy, Kaohsiung Medical University, No. 100, Shih-Chuan 1st Rd., Sanmin Dist., Kaohsiung 80708, Taiwan; hschang@kmu.edu.tw (H.-S.C.); m635013@kmu.edu.tw (I.-S.C.); 3 Department of Medical Research, Kaohsiung Medical University Hospital, No. 100, Tzyou 1st Rd. Sanmin Dist., Kaohsiung 80708, Taiwan; 4 Department of Cosmetic Science, Providence University, No. 200, Sec. 7, Taiwan Boulevard, Shalu Dist, Taichung 43301, Taiwan

**Keywords:** leukemia, ardisianone, tumor necrosis factor receptor 2, pyroptosis, differentiation

## Abstract

Acute myeloid leukemia (AML) is one of the most common forms of leukemia. Despite advances in the management of such malignancies and the progress of novel therapies, unmet medical needs still exist in AML because of several factors, including poor response to chemotherapy and high relapse rates. Ardisianone, a plant-derived natural component with an alkyl benzoquinone structure, induced apoptosis in leukemic HL-60 cells. The determination of dozens of apoptosis-related proteins showed that ardisianone upregulated death receptors and downregulated the inhibitor of apoptosis protein (IAPs). Western blotting showed that ardisianone induced a dramatic increase in tumor necrosis factor receptor 2 (TNFR2) protein expression. Ardisianone also induced downstream signaling by activating caspase-8 and -3 and degradation in Bid, a caspase-8 substrate. Furthermore, ardisianone induced degradation in DNA fragmentation factor 45 kDa (DFF45), a subunit of inhibitors of caspase-activated DNase (ICAD). Q-VD-OPh (a broad-spectrum caspase inhibitor) significantly diminished ardisianone-induced apoptosis, confirming the involvement of caspase-dependent apoptosis. Moreover, ardisianone induced pyroptosis. Using transmission electron microscopic examination and Western blot analysis, key markers including gasdermin D, high mobility group box1 (HMGB1), and caspase-1 and -5 were detected. Notably, ardisianone induced the differentiation of the remaining survival cells, which were characterized by an increase in the expression of CD11b and CD68, two markers of macrophages and monocytes. Wright–Giemsa staining also showed the differentiation of cells into monocyte and macrophage morphology. In conclusion, the data suggested that ardisianone induced the apoptosis and pyroptosis of leukemic cells through downregulation of IAPs and activation of caspase pathways that caused gasdermin D cleavage and DNA double-stranded breaks and ultimately led to programmed cell death. Ardisianone also induced the differentiation of leukemic cells into monocyte-like and macrophage-like cells. The data suggested the potential of ardisianone for further antileukemic development.

## 1. Introduction

Leukemia is one group of hematological malignancies originating in the blood and bone marrow. Many types of leukemia exist and occur when considerable abnormal white blood cells are generated to interfere with the normal function of bone marrow and blood cells. Symptoms of leukemia are diverse and may include bleeding and bruising, fever, persistent fatigue and weakness, weight loss, and severe infections. Some leukemic conditions are potentially life-threatening and need urgent and intensive treatment. However, the treatment of leukemia depends on the disease type and can be complicated. Chemotherapy is a major option for the treatment of leukemia. Side effects and insufficient efficacy always exist, however. Therefore, new therapeutic drugs and novel strategies are urgently needed [1,2,3]. Acute myeloid leukemia (AML) is one of the most common forms of leukemia. In spite of the advances in the management of such malignancies and the progress of novel therapies in recent decades, the overall outcome for patients with AML is still unsatisfactory for several possible reasons, such as poor response to chemotherapy and high relapse rates. Because of the substantial clinical and financial burdens, unmet medical needs still exist in such hematological malignancies [4].

Pyroptosis is a form of programmed cell death. Similarly to apoptosis, pyroptosis involves nuclear condensation, chromatin DNA fragmentation and positive staining with the TUNEL assay. During pyroptosis, pore formation on the cell membrane also disturbs the balance of ion gradients, leading to the release of several intracellular factors and proinflammatory mediators [5,6]. As a form of cell death, pyroptosis in tumors has become increasingly dominant in blocking the occurrence and development of tumors, particularly in antileukemic research [7,8]. Tumor necrosis factor (TNF) receptor 2 (TNFR2) plays dual roles in proinflammatory and anti-inflammatory activities and has shown protective effects on oligodendrocytes, cardiomyocytes, and keratinocytes [9]. TNFR2 has been suggested to mediate apoptosis directly or through a ligand-passing mechanism in which TNFR2 enhances TNF concentration in the vicinity of TNFR1, which receives a TNF ligand from TNFR2 and is activated, promoting TNFR1-induced apoptotic pathways [9,10,11,12]. In addition, TNFR2 has been suggested to mediate cell death through the cytoplasmic domain to trigger TNF expression and increase the TNFR1-mediated cell death program [10,13,14]. In recent decades, targeting of TNFR2 has attracted increasing interest for therapeutic approaches.

Plant-derived natural components are key resources capable of providing remarkable chemical diversity and biodiversity that can be used for medicinal chemistry and drug discovery. Ardisianone, a plant-derived natural component with an alkyl benzoquinone structure, can be derived from *Ardisia virens* Kurz (Myrsinaceae) [15] and *Ardisia compressa* tea extract [16] and has been demonstrated to display antiproliferative and apoptotic activities in cancer cells, such as those of hepatocellular carcinoma HepG2 and castration-resistant prostate cancer PC-3 and DU-145, through inhibition of the mTOR/p70S6K pathway [17]. However, the effect of ardisianone on the treatment of hematologic malignancies has not been delineated. In the current study, we evaluate the antileukemic effect and underlying mechanisms of ardisianone on HL-60, a cell model of acute promyelocytic leukemia (APL), a type of AML. The roles of several cellular proteins and both pyroptotic and apoptotic programmed cell death were studied to demonstrate the antileukemic potential of ardisianone.

## 2. Results

### 2.1. Ardisianone Induced a Cytotoxic Effect in HL-60 Cells

**Figure 1:**
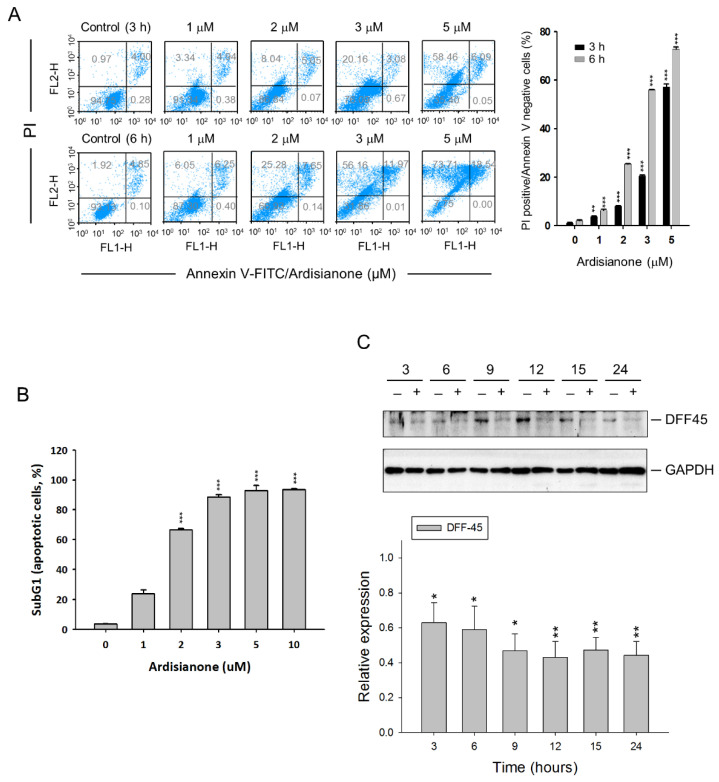
Determination of ardisianone-induced cell death in HL-60 cells. (**A**) Cells were treated with or without ardisianone at the indicated concentration and time. After the treatment, the cells were stained with Annexin V-PI to analyze apoptosis using flow cytometric analysis. (**B**) Cells were treated with graded concentrations of ardisianone for 24 h. After the treatment, the cells were fixed and stained with propidium iodide to analyze DNA content by FACScan flow cytometer, and quantitative analysis of sub-G1 (apoptosis) population was performed. (**C**) Cells were treated with or without ardisianone (2 μM) for 3 to 24 h. Total protein was extracted and subjected to SDS–PAGE for the detection of apoptotic DFF45/ICAD. Protein expression was quantified using Bio-Rad Image Lab^TM^ Software. * *p* < 0.05, ** *p* < 0.01, and *** *p* < 0.001 compared with respective controls.

The MTT colorimetric assay was used to evaluate the cytotoxic activity in HL-60 cells based on the reduction of a tetrazolium compound into an insoluble formazan compound by the mitochondria. Ardisianone displayed a cytotoxic activity with IC_50_ values of 1.87 and 1.67 μM after 24 and 48 h exposure to the cells, respectively. Annexin V/PI staining was subsequently used to examine cell death; it showed that ardisianone induced a time- and concentration-dependent increase in cell necrosis (annexin V negative/PI positive) and apoptosis (annexin V positive/PI positive) (Figure 1A). An ardisianone-mediated cytotoxic effect was substantiated by using flow cytometric analysis of PI staining, showing that ardisianone induced a concentration-dependent increase in the sub-G1 population (apoptosis) (Figure 1B). Furthermore, an inhibitor of caspase-activated DNase (ICAD), also known as DNA fragmentation factor 45 kDa (DFF45), was examined. The association of DFF45 with CAD inhibits DNase activity. DFF45 predominantly functions downstream of caspase-3. The cleavage of DFF45 can inactivate its inhibitory function on CAD, leading to DNA degradation by CAD and cell death [18]. Ardisianone induced a profound degradation of DFF45, indicating the involvement of caspase-dependent programmed cell death (Figure 1C). DAPI, which binds strongly to the minor groove of the adenine–thymine regions of DNA, was used to visualize nuclear change and to assess apoptosis. The data showed that ardisianone induced DNA fragmentation (Figure S1, Supplementary Materials). A single-cell comet assay also substantiated the DNA damage with the detection of comet tails (Figure S1, Supplementary Materials).

### 2.2. Ardisianone Induced Both Death Receptor- and Mitochondria-Mediated Programmed Cell Death

**Figure 2:**
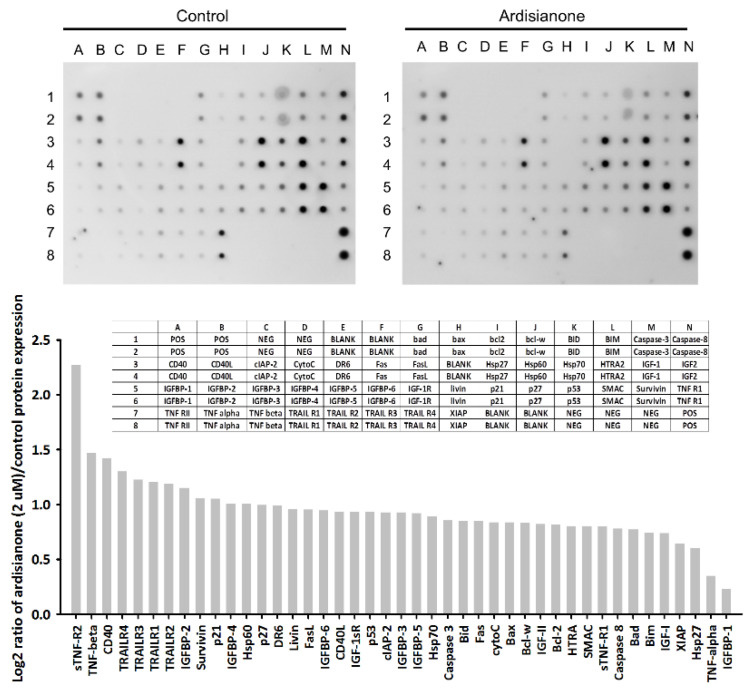
Protein array analysis of ardisianone-treated cells. HL-60 cells were incubated in the absence or presence of ardisianone (2 μM) for 9 h. The cells were lysed, and equal amounts of protein from each sample were used for the assay. Quantitative analysis in the arrays showed differences in the apoptotic marker proteins. All the protein levels were normalized in the positive dots.

**Figure 3:**
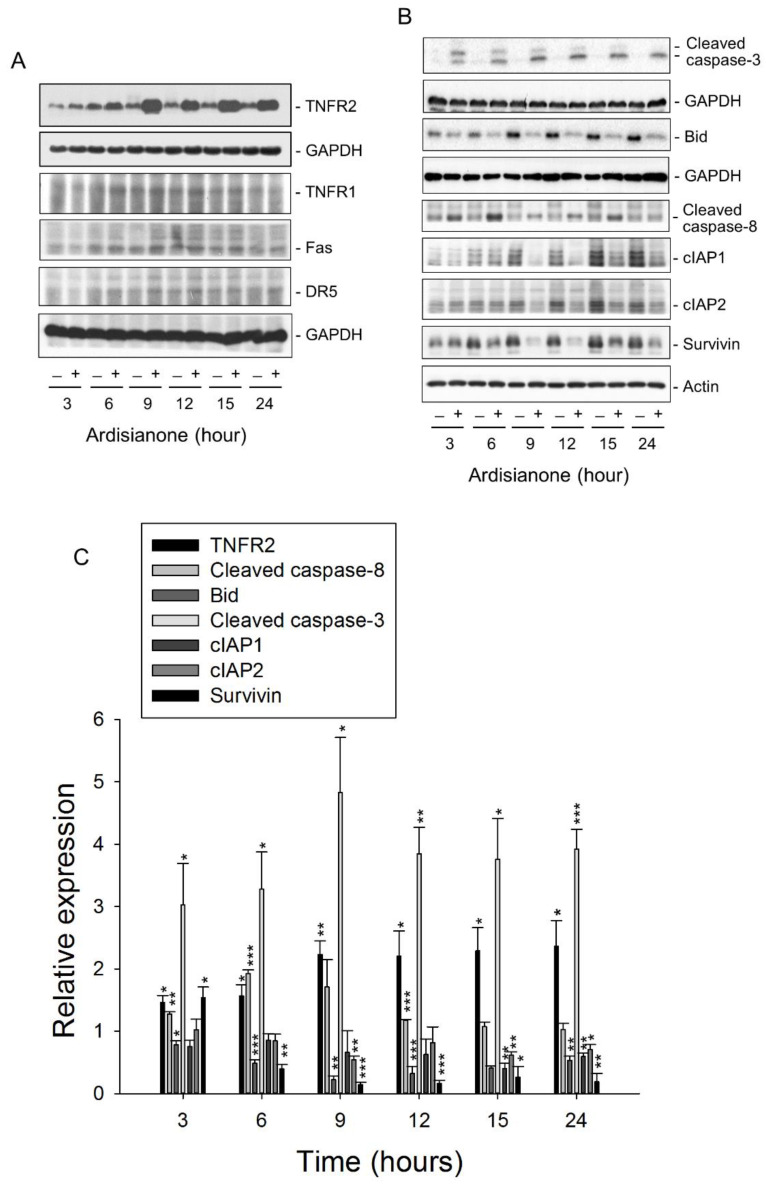
Effect of ardisianone on the expression of several proteins. HL-60 cells were incubated with or without ardisianone (2 μM) for the indicated times. After the treatment, the cells were harvested for the detection of protein expression of death receptors (**A**) and several proapoptotic and antiapoptotic protein expressions (**B**) using Western blotting analysis. (**C**) Expression levels were quantified using Image Lab Software 6.0 (BIO-RAD), and protein expression relative to ardisianone-free control was demonstrated. Data are expressed as mean ± SEM of three determinations. * *p* < 0.05, ** *p* < 0.01, and *** *p* < 0.001 compared with the control.

After the exposure of HL-60 cells to ardisianone, the cell lysates were used for the detection of 43 apoptosis-related proteins using a human apoptosis antibody array kit. As a result, the expression of several death receptors was upregulated, while that of inhibitors of apoptosis proteins (IAPs) were downregulated. TNFR2 was the most susceptible cellular target. Its protein expression was dramatically increased by ardisianone (Figure 2). The DAVID software was used to determine several functional classes based on ardisianone-regulated proteins. The top functional annotation clustering activated by ardisianone was the role of mitochondria in apoptotic signaling and apoptosis signaling in response to DNA damage (Figure S2, Supplementary Materials). Western blot analysis was further performed to substantiate the impact on death receptors. The results showed that ardisianone induced a time-dependent increase in the protein expression of TNFR2 but not that of TNFR1, Fas, or DR5 (Figure 3A,C). TNFR2 has been shown to trigger programmed cell death either directly or through enhancing TNFR1-induced apoptotic pathways [9,10,11,12]. The activation of caspase-8 was the major downstream event of the ligation between death ligands and their receptors. Once activated, caspase-8 is able to trigger two apoptosis pathways: one is the proteolytic activation of downstream executioner caspase-3 and -7; the other is the activation of the mitochondrial pathway mediated by the truncated Bid formed after the cleavage of Bid by caspase 8. Our data demonstrated that ardisianone induced the increased formation of both caspase-8 and -3 and degradation in Bid (Figure 3B,C).

**Figure 4:**
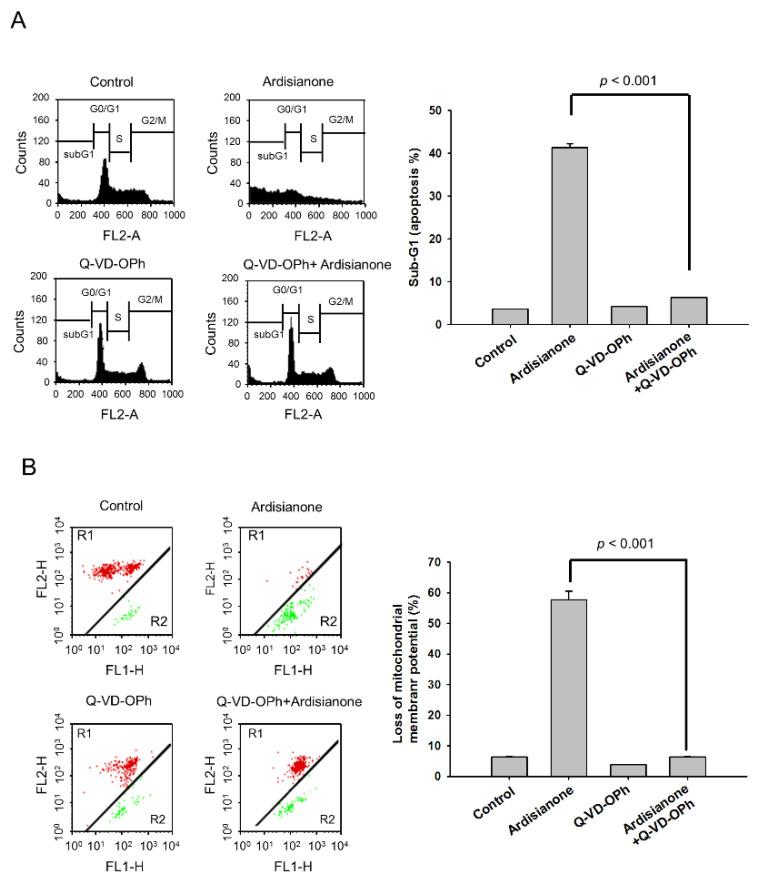
Effect of Q-VD-OPh on ardisianone-induced apoptosis and loss of mitochondrial membrane potential. HL-60 cells were treated in the absence or presence of the indicated agent (ardisianone, 2 μM; Q-VD-OPh, 20 μM) for 24 h. After the treatment, the cells were fixed and stained with propidium iodide to analyze DNA content (**A**) or incubated with JC-1 for the detection of mitochondrial membrane potential using flow cytometric analysis (**B**).

IAPs are a protein family that is frequently overexpressed in a variety of cancers and is responsible for cancer cell survival and chemoresistance through the control of ubiquitin-dependent signaling and negative regulation of caspases [19]. Increasing evidence shows that cIAP1 and cIAP2 are capable of binding to TNF receptor-associated factors (TRAFs) through their N-terminal baculovirus IAP repeat (BIR) motif-comprising domains to inhibit cell death induced by TNFR [20]. In contrast, survivin, which is the smallest member of the IAP family, can inhibit apoptosis through collaborative interactions with other IAP partners [21]. The data shown in Figure 3 demonstrated that ardisianone dramatically downregulated the protein expression of cIAP1, cIAP2, and survivin (Figure 3B,C), suggesting that ardisianone promoted cell death. Furthermore, Q-VD-OPh, a broad-spectrum caspase inhibitor, almost completely abolished the ardisianone-induced increase in the sub-G1 apoptotic population (Figure 4A) and prevented the loss of mitochondrial membrane potential (Figure 4B). These results confirmed the ardisianone-mediated caspase-dependent cell death and indicated that the mitochondrial damage was secondary to the caspase activation.

### 2.3. Ardisianone Induced Pyroptosis in HL-60 Cells

**Figure 5:**
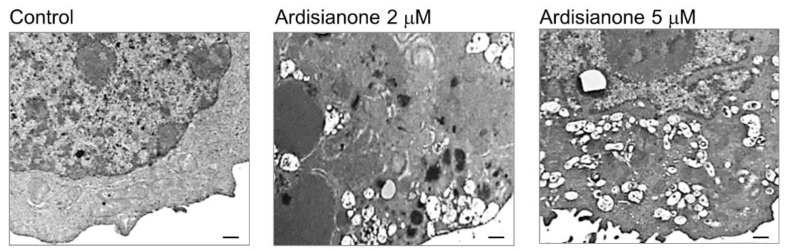
Transmission electron microscope examination of ardisianone-treated HL cells. The cells were treated with or without ardisianone (2 μM) for 24 h. The cells were harvested, washed, and fixed as described in the Materials and Methods section. The cells were dehydrated using alcohol and embedded in epoxy resin for sectioning. Ultrathin sections were stained with uranyl acetate and lead citrate and then examined with a Hitachi H-7500 transmission electron microscope. Scale bar, 500 nm.

Emerging evidence has suggested that pyroptosis, gasdermin-mediated programmed cell death, can be induced without any viral or bacterial infection but by chemotherapeutic agents including cisplatin, lobaplatin, doxorubicin, and paclitaxel [22]. Therefore, pyroptosis has drawn considerable attention in cancer research. Transmission electron microscopy (TEM) analysis was performed to study the ultrastructure and morphology of the cell. The control cells showed intact plasma membranes and nuclei, and the cytoplasm contained numerous organelles, of which the mitochondria and endoplasmic reticulum were the most apparent (Figure 5). When exposed to 2 μM ardisianone, the cells displayed a mixture of apoptosis (chromatin condensation and relocation) and pyroptosis (plasma membrane permeabilization, organelle swelling, and cytoplasmic granulation/vacuolization). Ardisianone at 5 μM induced extensive vacuolization and plasma membrane rupture and led to the cytoplasm lacking discernable organelles (Figure 5). However, the morphological hallmarks of pyroptosis are shared by necroptosis. Therefore, unique and distinct molecular mechanisms were required to characterize these programmed cell deaths.

**Figure 6:**
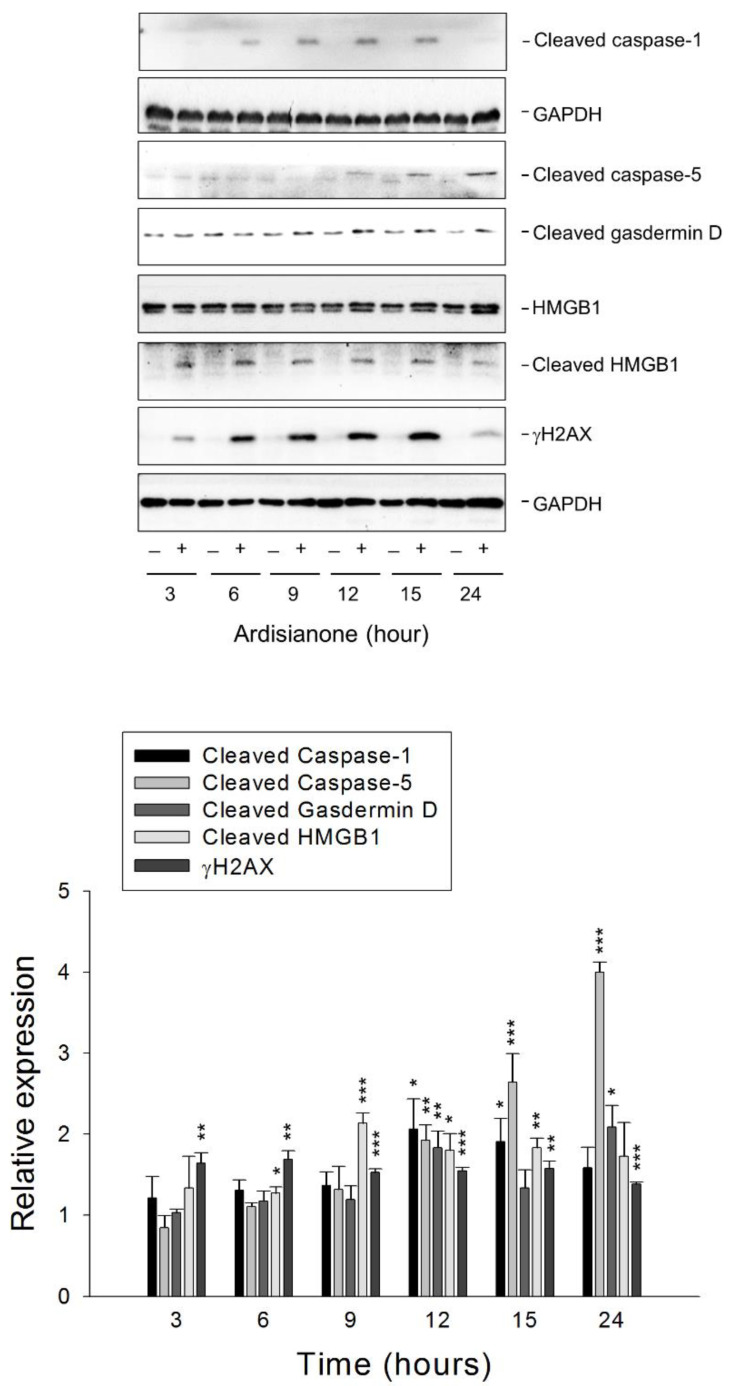
Effect of ardisianone on pyroptosis marker proteins. HL-60 cells were incubated with or without ardisianone (2 μM) for the indicated times. After the treatment, the cells were harvested for the detection of protein expression using Western blotting analysis. The expression levels were quantified using Image Lab Software 6.0 (BIO-RAD), and protein expression relative to ardisianone-free control was demonstrated. Data are expressed as mean ± SEM of three determinations. * *p* < 0.05, ** *p* < 0.01, and *** *p* < 0.001 compared with the control.

Pyroptosis is activated by human caspase-1, -4, and -5. Furthermore, it requires the cleavage and activation of gasdermin D, a pore-forming effector protein, by the caspases [23,24]. High mobility group box1 (HMGB1), a highly conserved nuclear protein, functions as an immune modulator and a DNA architectural factor. Pyroptosis has been shown to promote the release of HMGB1 [25]. Pyroptosis was detected using biochemical determination, showing that ardisianone induced the cleavage of both caspase-1 and -5 as well as gasdermin D. The protein expression of HMGB1 was moderately increased by ardisianone; in contrast, the cleavage of HMGB1 was significantly induced by ardisianone (Figure 6). Several cleavage sites have been identified within the HMGB1 structure for human neutrophil elastase, cathepsin G, and matrix metalloproteinase 3 using gel electrophoresis, Western blotting, and mass spectrometry [26]. These proteolytic enzymes, in particular neutrophil elastase and cathepsin G, are also widely involved in the processing of gasdermin D in the pyroptotic death program [27,28]. These results indicated the inducement of pyroptosis by ardisianone in HL-60 cells. It has been reported that chemotherapeutic agents can induce pyroptosis in cancer cells with impaired DNA damage repair or intensive DNA damage [29]. Further examination demonstrated that ardisianone induced a profound increase in phosphorylation of H2AX at ser139 (γ-H2AX), a sensitive marker of DNA double-stranded breaks (Figure 6). We also employed immunofluorescence to examine the coexpression of γ-H2AX and Rad51 foci formation (a marker of DNA damage repair). We detected strong positive staining for γ-H2AX and Rad51 foci formation, indicating substantial DNA damage with concomitant DNA repair activity. However, the cells eventually underwent apoptosis, as evidenced by DNA fragmentation (fragmented DAPI staining) (Figure S3, Supplementary Materials). We assessed the colocalization of γ-H2AX and Rad51 foci staining in more than 100 cells. The data showed that 50.7% of Rad51 foci colocalized with the γ-H2AX label for HL-60 cells.

### 2.4. Ardisianone Induced Differentiation of HL-60 Promyelocytes

**Figure 7:**
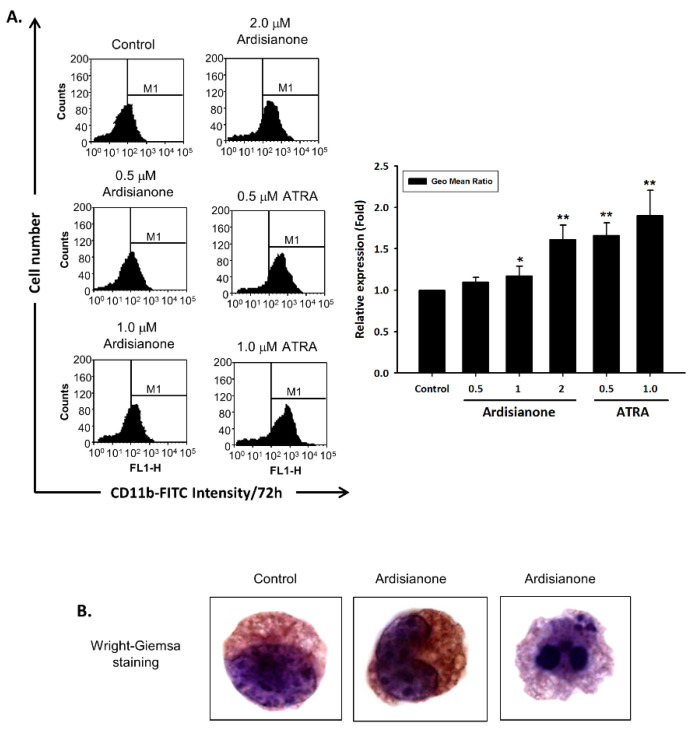
Effect of ardisianone on cell differentiation. HL-60 cells were incubated in the absence or presence of ardisianone or all-*trans*-retinoic acid (ATRA) at the indicated concentration (**A**) or in 2 μM ardisianone (**B**) for 72 h. Then, the cells were harvested and either incubated with FITC-conjugated anti-CD11b antibody and analyzed by flow cytometry (**A**) or stained with Wright–Giemsa stain solution (**B**) and examined under a light microscope BX56 (Olympus, Japan). * *p* < 0.05 and ** *p* < 0.01 compared with the control.

A characteristic feature of leukemia cells is the inhibition of differentiation at certain stages of cellular maturation. The most notable success in the treatment of APL, treatment with all-*trans*-retinoic acid, has encouraged the continued development of differentiation therapy in antileukemia strategies. In addition to the inducement of both apoptotic and pyroptotic cell death, ardisianone resulted in the significant differentiation of the remaining survival cells, as identified by an increased expression of CD11b (Figure 7A) and CD68 (Figure S4, Supplementary Materials), two differentiation markers for cells of the monocyte/macrophage lineage. Wright–Giemsa staining was performed to study the morphological changes of the cells. The control cells were observed with a lymphoblastic morphology. Exposure to ardisianone (2 μM) for 72 h induced the differentiation of the cells into monocyte and macrophage morphologies (Figure 7B), confirming the differentiation capability of ardisianone.

## 3. Discussion

Leukemias account for a considerable percentage of cancers worldwide. There exist therapeutic challenges in treating leukemias because of their heterogeneity and biological features. Novel therapeutic approaches are therefore necessary to improve treatment outcomes. The utilization of natural products and their novel structures is a key reservoir for discovering and developing therapeutic drugs, particularly in the area of cancer research. More than half of the small molecules approved by the FDA in cancer therapy are natural products, derivatives thereof, or compounds from numerous natural product structures [30]. Ardisianone is a natural product with an alkyl benzoquinone structure. We focused on the discovery and biological evaluation of ardisianone using the APL cell model. The data demonstrated the cytotoxic activity of ardisianone with a dramatic inducement of TNFR2 protein expression. TNFR2 has been proven to elicit anti-inflammatory activities in immune cells, although its proinflammatory effects have also been shown [9]. Notably, TNFR2 has been suggested to regulate Fas ligand/Fas and TNF/TNFR1-mediated cell death signaling [31]. A ligand-passing pathway of TNFR2 has been reported to increase TNF levels in TNFR1 by receiving TNF ligands from TNFR2. This pathway may enhance TNFR1-mediated apoptotic signaling [9,10,11,12]. Furthermore, Aguadé-Gorgorió and colleagues reported that the mRNA expression of TNFR2 positively correlated with the antileukemic activity of birinapant and LCL161, two synthetic small molecules of second mitochondrial-derived activators of caspases (SMAC) and inhibitors of IAP family proteins. This cytotoxic effect was examined to give a TNF-α-independent mechanism [32]. Our data supported the possibility of TNFR2-involved mediation of antileukemic activity.

The involvement of TNFR2 in TNF-induced programmed cell death is complex; it may cooperate with TNFR1 or mediate the cell death in a direct manner as discussed below [33,34]. The initiation of TNF/TNFR1 activation signaling may trigger the recruitment of the complex consisting of TNFR-associated death domain (TRADD), RIP1, TRAF2, TRAF5, and cIAPs, where the cIAPs catalyze RIP1 into a polyubiquitinated form for the binding of transforming growth factor β-activated kinase 1 (TAK1), TAK1-binding protein2 (TAB2), and TAB3. The TAK1–TAB2–TAB3 complex activates the classical NF-κB pathway to induce the transcription of cytoprotective genes for cell survival [33,35]. TNFR2 activation has been shown to recruit TRAF2–cIAP1/2 and TRAF1–TRAF2–cIAP1/2 complexes to TNFR2, leading to a profound depletion of these cytosolic complexes and promoting TNFR1-dependent caspase-8 activation and apoptosis [33,36]. In contrast, Depuydt and colleagues reported that TNFR2 activation did not enhance the susceptibility of TNFR1-induced apoptotic signaling. Although TRAF2 downregulation has been proposed to be responsible for the mechanism by which TNFR2 sensitizes TNFR1 signaling, it did not modulate TNFR1-induced effects. Depuydt et al. further identified that, similarly to TNFR1, TNFR2 utilizes FADD to activate caspase-8 and death-inducing pathways [34]. Our data showed that ardisianone dramatically upregulated TNFR2 but downregulated both cIAP1 and cIAP2. In light of the key role of TRAF2–cIAPs complexes in TNFR1-mediated cell survival (e.g., classical NF-κB signaling), the downregulation of cIAPs supported the crosstalk between TNFR1 and TNFR2 in ardisianone-induced programmed cell death. However, it is noteworthy that cIAPs are also the substrates for caspase-8 [37]. Because ardisianone induced the activation of caspase-8, the downregulation of cIAPs could be attributed to caspase-8 activation, which supports the possibility that TNFR2 triggered cell death in a TNFR1-independent pathway. However, the signaling complexes involved in ardisianone-induced cell death remain elusive and require further investigation.

Notably, ardisianone induced caspase-dependent mitochondrial damage upon complete rescue by a broad-spectrum caspase inhibitor. Caspase-8 activation could be responsible for the mitochondrial damage, since the caspase-8 substrate Bid, a Bcl-2 family member engaging in mitochondrial function, was substantially degraded. However, several studies indicated that caspase-dependent apoptosis could not fully explain cell death in the TNF death pathway [38]. Our data also showed that, in addition to apoptosis, ardisianone induced a substantial and time-dependent programmed cell necrosis. Necroptosis is a major form of regulated necrotic cell death in response to death receptor signaling. Predominantly regulated by receptor-interacting protein 1 (RIP1), RIP3, and mixed-lineage kinase domain-like protein (MLKL), necroptosis can function as an alternative programmed cell death to overcome apoptosis resistance [39]. The collaboration between TNFR1 and TNFR2 has been reported to trigger cell necroptosis [33,40]. Notably, TNF-mediated necroptosis can occur only when caspase-8 is inhibited or deficient, since caspase-8 plays a crucial role in preventing necroptosis [33,41]. As such, ardisianone-induced programmed necrotic cell death during caspase-8 activation was probably not necroptosis. Further identification showed that necrostatin-1, a specific necroptosis inhibitor targeting the death domain of RIP1, failed to rescue ardisianone-induced cell death (unshown data), confirming a cell death other than necroptosis. Pyroptosis is another form of regulated necrotic cell death and is typically triggered by the activation of caspase-1, -4, and -5 in humans, leading to the cleavage of gasdermin D, pore formation, and cell lysis [23,24,41]. Numerous studies have revealed coincident signaling between caspase-8 activity and gasdermin D-involved necrotic cell death [41,42]. Moreover, it has been shown that gasdermin D is cleaved by purified active caspase-8 [42]. These studies provide evidence that caspase-8 can cause the onset of pyroptosis. Our data revealed synchronicity between the activation of caspase-1, -5, and -8 and the cleavage of gasdermin D, suggesting the key role of the caspase activation pathway in ardisianone-induced pyroptosis.

Conquering the block to myeloid differentiation in acute myeloid leukemia (AML) is an important therapeutic approach in which the discovery of all-*trans*-retinoic acid (ATRA) has been the most remarkable success. Accordingly, numerous small molecules have been developed as differentiation strategies. However, the next task is to discover the protein targets of the agents responsible for the differentiation activity. HL-60 promyelocytic leukemia cells have been extensively used to study granulocytic differentiation. ATRA-induced granulocytic differentiation can be substantiated by both the identification of nuclear morphology and changes in CD11b expression [43]. Similarly to ATRA, ardisianone induced the differentiation of the remaining survival cells into both monocyte-like and macrophage-like morphologies, as evidenced by the identification of nuclear morphology and increased expression of both CD11b and CD68. It has been shown that TNF-α enhances the differentiation of myeloid leukemia cells along a monocyte/macrophage pathway [44,45]. TNF-α predominantly mediates a survival/differentiation pathway through TNFR2 [46], which may be attributed to the inducement of the nuclear translocation of NF-κB, which in turn leads to the activation of target genes [45,47]. Witcher and colleagues reported that ATRA promoted TNF-α-induced target genes in monocytic differentiation through chromatin remodeling to enhance the binding of transcription factors (e.g., NF-κB) to the response elements [45,48]. Our data supported the possible role of TNFR2 in ardisianone-induced differentiating activity, although the cellular targets need further elucidation.

In summary, the data suggested that ardisianone induced both the apoptosis and pyroptosis of leukemic cells through substantial upregulation of TNFR2 expression and activation of the caspase-1/-5/-8 pathways. Ardisianone also induced mitochondrial damage, gasdermin D cleavage, and DNA double-stranded breaks that ultimately led to programmed cell death. The remaining survival cells after ardisianone challenge were differentiated into monocyte-like and macrophage-like cells. The data suggest the potential of ardisianone for further antileukemic development.

## 4. Materials and Methods

### 4.1. Chemicals, Reagents, and Cell Culture

Methylthiazolyldiphenyl-tetrazolium bromide (MTT), dimethyl sulfoxide (DMSO), and propidium iodine (PI) were purchased from Sigma-Aldrich (St. Louis, MO, USA). JC-1 (5,5′,6,6′-tetrachloro-1,1′,3,3′ Tetra- ethylbenzimidazolylcarbocyanine iodide) was obtained from Molecular Probes (Invitrogen, Karlsruhe, Germany). Q-VD-OPh was purchased from R&D (Minneapolis, MN, USA). The antibodies of GAPDH, actin, PARP-1, and Rad51were obtained from Santa Cruz Biotechnology, Inc. (Santa Cruz, CA, USA). Antibodies of DFF45/DFF35, TNFR1, TNFR2, Fas, DR5, Bid, cleaved caspase-1, caspase-3, caspase-5, caspase-8, gasdermin D, cIAP1, cIAP2, survivin, HMGB1, and γH2A.X^Ser139^ were obtained from Cell Signaling Technologies (Boston, MA, USA). Antimouse and antirabbit IgGs were obtained from Jackson Immuno-Research Laboratories, Inc. (West Grove, PA, USA). HL-60 (promyelocytic leukemia) was obtained from Bioresource Collection and Research Center (BCRC, Hsinchu, Taiwan). HL-60 cells were cultured in Iscove’s Modified Dulbecco’s Medium (IMDM) (Gibco, Grand Island, NY, USA) with 10% FBS (*v*/*v*) and penicillin (100 U/mL)/streptomycin (100 mg/mL). The cells were grown in a water-saturated atmosphere at 5% CO_2_ and at 37 °C.

### 4.2. Cell Viability Assay

Cells, at a density of 3 × 10^5^ cells/mL, were cultured in a 24-well plate. After the treatment of cells with compound at the indicated concentrations and times, an assay for mitochondrial MTT reduction activity was performed. MTT was dissolved in phosphate-buffered saline (PBS) at a concentration of 5 mg/mL and filtered. From the stock solution, 50 μL per 500 μL of medium was added to each well, and plates were gently shaken and incubated at 37 °C for 2 h. After the loading of MTT, the medium was replaced with 1 mL 100% DMSO and was left for 5–10 min at room temperature for color development. The plate was read by an enzyme-linked immunosorbent assay reader (570 nm) to get the absorbance density values.

### 4.3. Flow Cytometric Detection of Apoptosis

After the treatment, the cells were harvested, washed twice with ice-cold PBS, fixed with 70% ethanol at 4 °C for 30 min, and washed again with ice-cold PBS. The cells were then resuspended with 0.2 mL of PI solution containing Triton X-100 (0.1% *v*/*v*), RNase (100 mg/mL), and PI (80 mg/mL) in the dark. Cells were analyzed with FACScan™ and CellQuest™ software (Becton Dickinson, Mountain View, CA, USA). The level of apoptotic cells containing sub-G1 DNA content was determined as a percentage of the total number of cells. In another assay, the cells were stained with FITC-Annexin V/PI using an apoptosis detection kit (BD Pharmingen) according to the manufacturer’s protocol. After incubation at room temperature for 15 min in the dark, apoptosis was detected and quantified using flow cytometric analysis.

### 4.4. Transmission Electron Microscopy

HL-60 cells (5 mL of 3 × 10^5^ cells/mL) were treated with or without 2 μM ardisianone for 24 h. Cells were harvested and washed with 0.1 M PBS. The cell pellets were fixed in 2.5% glutaraldehyde and postfixed in 1% osmium tetroxide and were then dehydrated using alcohol and embedded in epoxy resin for sectioning. Ultrathin sections were stained with uranyl acetate and lead citrate and then examined with a Hitachi H-7500 transmission electron microscope (TEM).

### 4.5. Measurement of Mitochondrial Membrane Potential (ΔΨm)

JC-1 was used to determine ΔΨm. Cells were treated in the absence or presence of the indicated agent. Thirty minutes before the termination of incubation, the cells were incubated with JC-1 (final concentration of 2 μM) at 37 °C for 30 min. The cells were finally harvested, and the accumulation of JC-1 was determined using flow cytometric analysis.

### 4.6. Human Apoptosis Antibody Array

To investigate the pathways by which ardisianone induces apoptosis, we performed a determination of apoptosis-related proteins using an antibody array (human apoptosis antibody array kit, Raybiotech, Norcross, GA, USA) according to the manufacturer’s instructions. After treatment with ardisianone, the cells were collected, and 300 μg of protein extract from each sample was incubated with the antibody array membrane for 4 h. The membrane was quantified using a Biospectrum AC ChemiHR 40 system (UVP, Upland, CA, USA), and the membrane image file was analyzed using UVP analysis software.

### 4.7. Protein Expression Network Construction and Functional Annotation Analysis

A list of ardisianone-modulating proteins was uploaded to the Database for Annotation, Visualization, and Integrated Discovery (DAVID) bioinformatics resource to investigate the biological networks associated with these proteins (https://david.ncifcrf.gov/, accessed on 11 August 2019). Functional annotation of the modulating proteins was carried out using David functional annotation clustering. This analysis used computational algorithms to identify networks consisting of focus proteins (proteins that were present in our list) and their interactions with other proteins (“nonfocused”) in the knowledge base. Scores were calculated for each network according to the fit of the network to the set of focus proteins and used to rank networks in the DAVID bioinformatics analysis. The functional enrichment analysis based on each interested module was finished within DAVID by functional annotation pathway analysis.

### 4.8. Western Blotting

After the treatment, cells were harvested, centrifuged, and lysed in 0.1 mL of lysis buffer containing 20 mM Tris-HCl (pH 7.4), 150 mM NaCl, 1% Triton X-100, 1 mM EDTA, 1 mM PMSF, 1 μg/mL leupeptin, 1 mM NaF, 1 mM sodium orthovanadate, and 1 mM dithiothreitol. Total protein was quantified, mixed with sample buffer, and boiled at 95 °C for 5 min. An equal amount of protein (30 μg) was separated by electrophoresis in a 12% SDS–PAGE, transferred to PVDF membranes, and detected with specific antibodies. The immunoreactive proteins, after incubation with appropriately labeled secondary antibody, were detected with an enhanced chemiluminescence detection kit (Amersham, Buckinghamshire, UK).

### 4.9. Cytological Morphology Staining

After the treatment, the cells were collected by centrifugation and resuspended in 200 μL of PreservCyt^®^ solution (Hologic, Inc., Marlborough, MA, USA). The suspension was passed through a Thinprep^®^ (Hologic, Inc., Marlborough, MA, USA) processing machine, and the cells were collected. The slides were fixed in 95% alcohol and then stained with Wright–Giemsa (Sigma Diagnostics, Livonia, MI, USA) for 5 min at room temperature. Stained cells from each treatment group were examined under a light microscope BX56 (Olympus, Tokyo, Japan).

### 4.10. Alkaline Comet Assay

Single-cell comet assays were performed using the Trevigen CometAssay^®^ Kit (Trevigen, Gaithersburg, MD, USA) with modifications. After treatment, the cells were collected by centrifugation and resuspended in cold Ca^2+^-free phosphate-buffered saline (1× PBS), mixed with low-melt agarose (1:10 ratio), and spread on frosted-glass slides. After the agarose solidified, the slides were sequentially placed in lysis and alkaline solutions. Slides were then subjected to electrophoresis at 25 mA for 5 min in 1× Tris-borate-EDTA (TBE) buffer. After that, the slides were stained with 50 μL of diluted SYBR^®^ Green I Nucleic Acid Gel Stain (Thermo Fisher Scientific, Waltham, MA, USA) completely before viewing. The slides were examined by using a BX56 fluorescent microscope (Olympus, Japan) with magnification 200×, and the images were captured.

### 4.11. Immunohistochemistry and Immunofluorescence Staining

After the treatment, the cells were collected by centrifugation and resuspended in 200 μL of PreservCyt^®^ solution (Hologic, Marlborough, MA, USA). The suspension was passed through a Thinprep^®^ processing machine, and the cells were collected. A standard immunohistochemistry (IHC) protocol using the DAKO EnVision™ staining kit (DAKO, Carpinteria, CA, USA) was used for IHC reactions according to the manufacturer’s instructions. The slides were fixed in 95% alcohol, rinsed in 1× Tris-buffered saline (TBS), and then treated with 3% H_2_O_2_ to inactivate endogenous peroxidase. The slides were then treated with Dako Antibody Diluent to block nonspecific binding. For immunohistochemistry staining, after blocking, the slides were stained with the mouse monoclonal antibody to RIP1 (Santa Cruz Biotechnology, Dallas, TX, USA) overnight at 4 °C. Washing steps in between were performed in TBS that contained 0.05% Tween 20 as the detergent. Hematoxylin (Merck, Germany) was used for background staining. For immunofluorescence staining, slides were then incubated with specific antibodies overnight at 4 °C. Next, the slides were rinsed and incubated with fluorescent secondary antibodies and ready-to-use DAPI (Santa Cruz Biotechnology, Dallas, TX, USA) for 3 min at room temperature. Finally, slides were covered with a glass coverslip. IHC and immunofluorescent stained slides were imaged with a BX56 light/fluorescent microscope (Olympus, Japan). All images were digitally acquired at 1000× magnification.

### 4.12. Data Analysis

Experimental data are presented as the mean ± standard error of the mean (SEM) of three to five independent experiments. Normality and homogeneity of variance assumptions were checked. The statistical analysis was performed using unpaired *t* tests. *p* values less than 0.05 were considered statistically significant. The Statistical Package for the Social Sciences version 12.0 (SPSS Inc., Chicago, IL, USA) software and the GraphPad Prism version 3.0 (GraphPad Software Inc., La Jolla, CA, USA) software were used.

## Data Availability

Not applicable.

## Supplementary Materials

The following are available online at https://www.mdpi.com/article/10.3390/ijms222011239/s1, Figure S1: Determination of ardisianone-induced cell death in HL-60 cells, Figure S2: Functional annotation analysis from protein array analysis in ardisianone-treated cells, Figure S3: Effect of ardisianone on DNA damage signals, Figure S4: Effect of ardisianone on cell differentiation.

## Abbreviations

AML	Acute myeloid leukemia
APL	Acute promyelocytic leukemia
ATRA	All-*trans*-retinoic acid
BIR	Baculovirus IAP repeat
DFF	DNA fragmentation factor
DMSO	Dimethyl sulfoxide
HMGB1	High mobility group box1
IAP	Inhibitor of apoptosis protein
ICAD	Inhibitor of caspase-activated DNase
IHC	Immunohistochemistry
MLKL	Mixed-lineage kinase domain-like protein
MTT	Methylthiazolyldiphenyl-tetrazolium bromide
PBS	Phosphate-buffered saline
PI	Propidium iodine
RIP	Receptor-interacting protein
TAB	TAK1-binding protein
TAK	Transforming growth factor β-activated kinase
TEM	Transmission electron microscopy
TNFR	Tumor necrosis factor receptor
TRADD	TNFR-associated death domain
TRAF	TNF receptor-associated factor
