# Supplementary figures and images for: Antileukemic Natural Product Induced Both Apoptotic and Pyroptotic Programmed Cell Death and Differentiation Effect
Source: Int J Mol Sci. 2021 Oct 18;22(20):11239. doi: 10.3390/ijms222011239 (PMC8538678; doi:10.3390/ijms222011239)

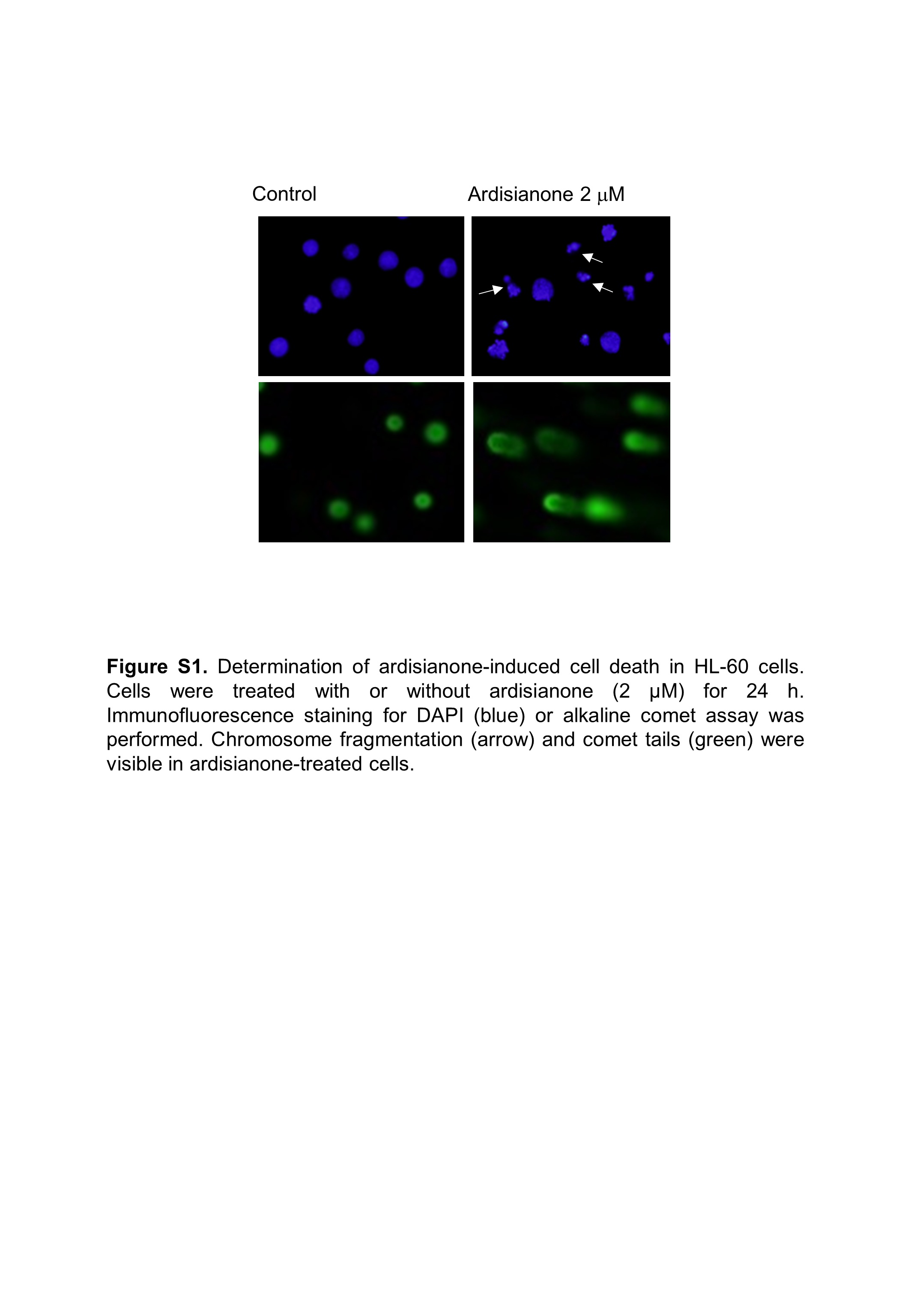

Supplement: Supplementary file 1 [file ijms-22-11239-s001.zip › Figure S1.tif]

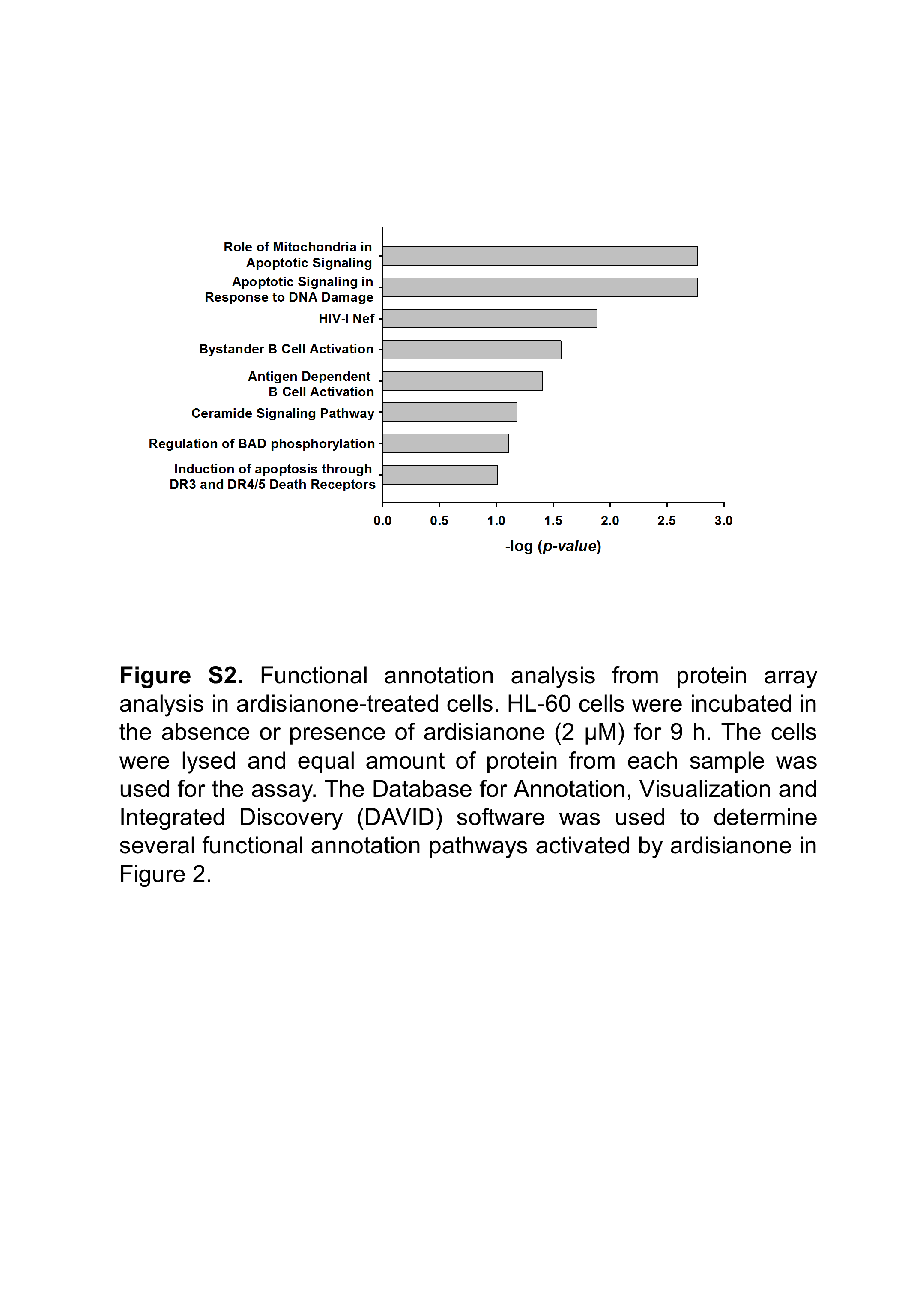

Supplement: Supplementary file 1 [file ijms-22-11239-s001.zip › Figure S2.tif]

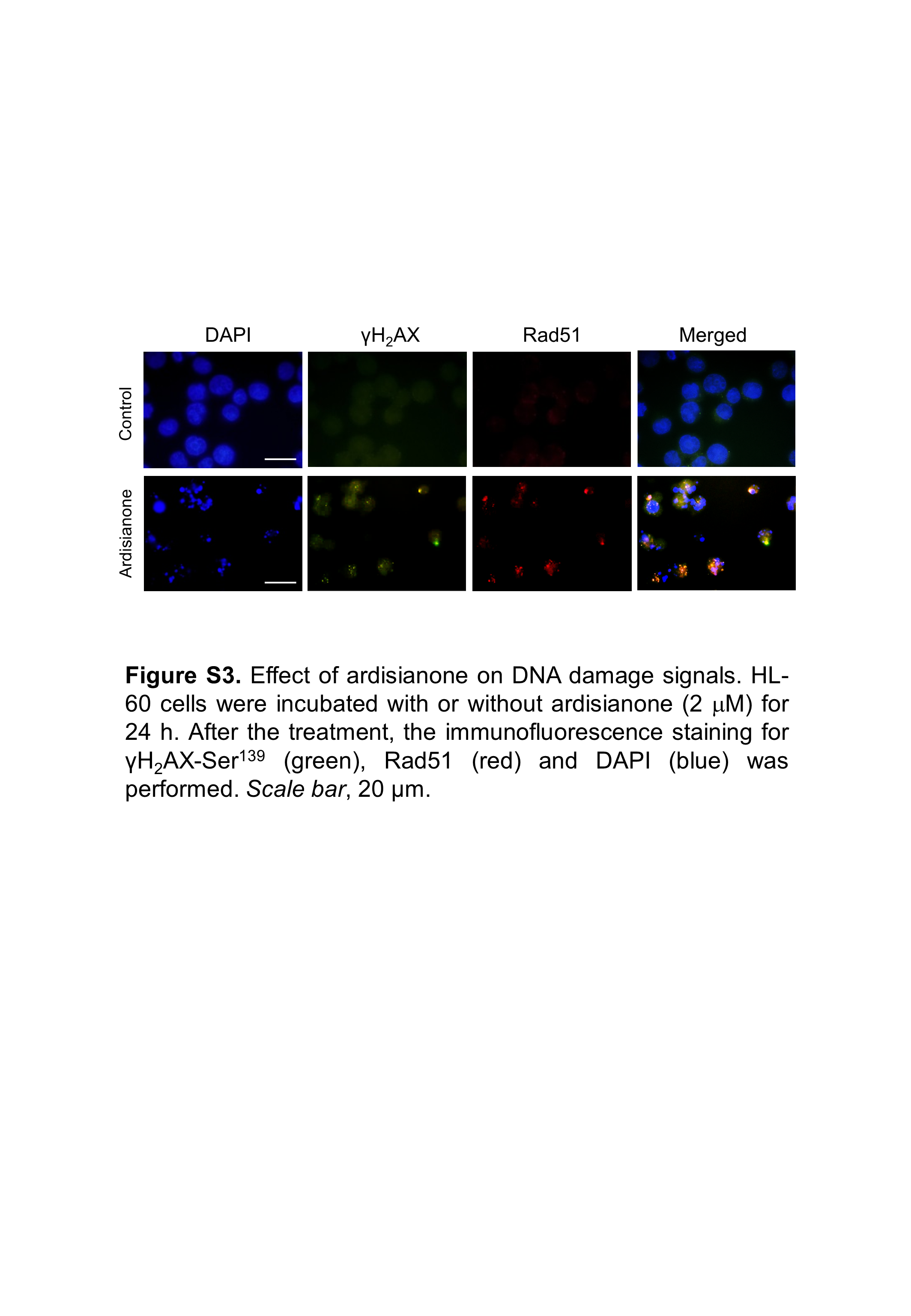

Supplement: Supplementary file 1 [file ijms-22-11239-s001.zip › Figure S3.tif]

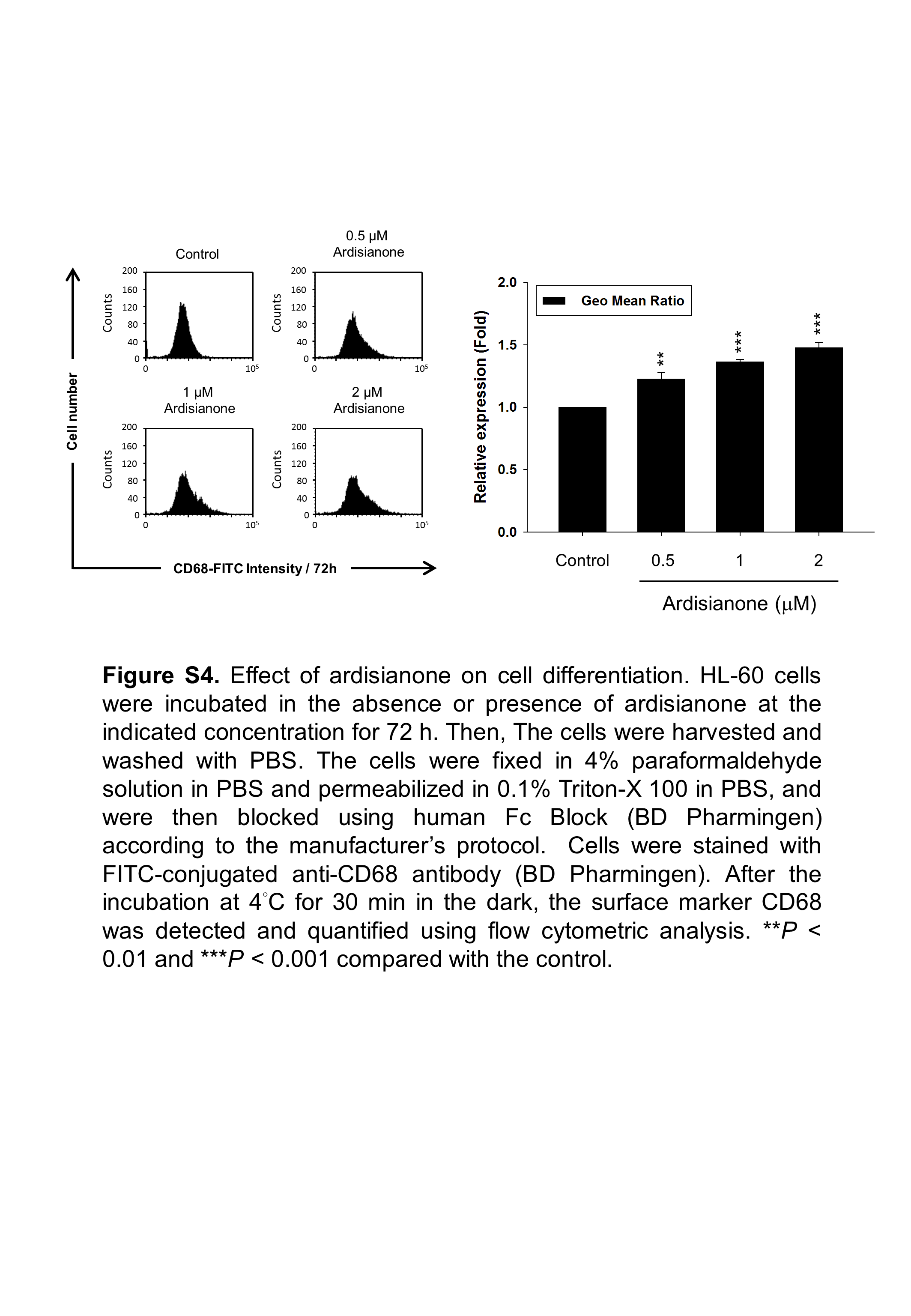

Supplement: Supplementary file 1 [file ijms-22-11239-s001.zip › Figure S4.tif]
